# Assessment of Respiratory Symptoms and Pulmonary Function Status among Workers of Flour Mills in Addis Ababa, Ethiopia: Comparative Cross-Sectional Study

**DOI:** 10.1155/2018/9521297

**Published:** 2018-10-24

**Authors:** Dessalegn Demeke, Diresibachew W. Haile

**Affiliations:** ^1^Bahir Dar University College of Medicine and Health Sciences, Department of Physiology, Bahir Dar, Ethiopia; ^2^Addis Ababa University College of Medicine and Health Sciences, Department of Physiology, Addis Ababa, Ethiopia

## Abstract

**Background:**

Flour dust in the respiratory tract affects lung function. Flour dust is a heterogeneous organic substance which can have a tendency to cause respiratory ailments. There is growing consensus on the deleterious effects of flour dust on respiratory symptoms and lung performance of flour mill workers.

**Methods:**

The study design was comparative cross-sectional. A total of 54 flour mill workers who work for more than eight-hour shift per day and 54 control subjects matched for sex, age, weight, height, and area of residence were enrolled. Anthropometric measurement was done. Lung function was measured by using a digital portable spirometer (Spiro Pro) based on the ATS guidelines. FVC, FEV_1_, FEV_1_/FVC, PEFR, and FEF_25_%  _−75_% were measured. Productive cough, dry cough, wheeze, and breathlessness were evaluated using BMRC questionnaire guidelines, administered through face-to-face interview.

**Result:**

This study showed statistically significant reduction in the mean values of pulmonary function tests in flour mill workers as compared to their matched controls. Reduction of pulmonary function indices in study subjects was significant for FVC (4.25±0.93 vs. 5.30±0.71, p<0.001), FEV_1_ (3.46±0.86 vs. 4.50±0.72, p<0.001), PEFR (5.43±2.43 vs. 7.87±2.53, p<0.001), and FEF_25_%  _−75_%, (3.87±1.61 vs. 4.60±1.60, p<0.05), but not significant for FEV_1_/FVC (81.93±12.74 vs. 83.40±12.50, p>0.05). Flour mill workers developed 27.7% of restrictive type and 11.1% of obstructive type of lung disorders. Percentage prevalence of respiratory symptoms was evaluated as dry cough (27.7% vs. 9.3%), productive cough (11.1% vs. 5.6%), wheeze (14.8% vs. 3.8%), and breathlessness (16.6% vs.7.4%) in flour mill workers and controls, respectively.

**Conclusion:**

Based on the results of the present study, occupational exposure to flour dust could cause respiratory dysfunction, thereby reducing lung efficiency.

## 1. Background

Industrialization has been established to optimally fulfill various human needs. Over the course of time, some industries exert hazardous effect on the health of human beings [1]. Those hazard effects do have significant variations in occupational health and safety performance between countries and economic sectors. Occupational hazards and the health problems associated with occupational health and safety follow different approaches like environmental assessment, biological monitoring, medical surveillance, and epidemiological approaches to identify health problems [2].

Occupational lung diseases are a broad group of diagnosis caused by the inhalation of dusts and chemicals. As a result, improper control of these substances can result in a severe threat to site workers because most of the workers are unaware of this hazardous effect on health [3]. According to NIOSH, atmospheric pollutants, like particulates, are capable of producing adverse effects under some condition(s) of exposure and dose through time [4]. Airborne dusts are particular concern to health, as they are to be associated with occupational lung diseases [5] and exposure to flour dust occurs across a range of food industries including grain mills, flour mills, and bakeries [6, 7].

Flour dust is a heterogeneous substance with respiratory sensitizing properties and long-term exposure to it may cause acute or chronic respiratory disease [8]. Several studies showed that prevalence of sensitization for wheat allergens and fungal a-amylase and OAD is high among workers exposed to flour dust [9]. This can have a tendency to cause respiratory ailments [10]. Poor ventilation is a basic problem in flour mills [11] that have continuous exposure of workers. There are more than 250 million work-related accidents every year [2, 6]. Agriculture, basic food processing, and extractive industries are very widespread and can lead to dust exposure. In many countries, several studies investigated pulmonary function and respiratory symptoms in occupational workers worldwide [11–13]. In Ethiopia, there was no documented data related to the effect of flour dust on pulmonary function among flour mill workers.

The study aims to investigate the effect of flour dust on the pulmonary function by analyzing the pulmonary function tests that will estimate the effectiveness of the lung. This study is therefore undertaken to assess the respiratory symptoms and pulmonary function problems related to flour mill workers. It will also encourage communication between various concerned groups and organizations and foster an improved understanding of the potential problems of flour dust in the country.

## 2. Methods

### 2.1. Study Designs

Comparative cross-sectional study design was employed to assess respiratory symptoms and pulmonary function parameters in workers of flour mills. The study was conducted in Lideta subcity, Addis Ababa, Ethiopia, from August to September 2016. Addis Ababa encompasses 10 subcities and 116 woredas. It is located at altitude of 2,400 meters above sea level. Based on the data from Lideta subcity trade and development bureau and urban agriculture corporation, ten of 116 woredas are located in Lideta subcity containing a total of 76 flour mills during a time of data collection.

### 2.2. Sampling Methods

The source populations were all flour mill workers in Addis Ababa city, Ethiopia. The study population were all flour mill workers and controls in Lideta subcity in the age range between 18 and 43 years.

**Study group: **it consisted of flour mill workers in Lideta subcity in the age range between 18 and 43 years. All subjects were male as there were no female workers in the flour mill industry in the selected sites during the study period.

**Control group**: non-flour mill workers in Lideta subcity anthropometrically matched to study group were selected as control group.

Double proportion formula was used for the determination of sample size. The proportions of the sample size were calculated from previous study on association of flour dust on PFTs and prevalence of respiratory symptoms of chronic cough for flour mill workers and control groups (37% vs. 13%, respectively) [14]. Then, the following assumption was used: confidence level 95% (1.96), 80% power of test, correction formula was applied then after considering nonresponse rate of 10%, and the final sample size was one hundred and eight.

### 2.3. Eligibility Criteria 

#### 2.3.1. Inclusion Criteria

Millers who were available in the workplace between the age range of 18 and 64 years and working in the flour mill for more than one year were included.

#### 2.3.2. Exclusion Criteria

Subjects with gross clinical abnormalities of the vertebral column, thoracic cage, neuromuscular diseases, known cases of gross anemia, diabetes mellitus, pulmonary tuberculosis, hypertension, drug addicts, known cardio pulmonary disease, cigarette smokers, tobacco and khat chewers, bakery workers, and those who underwent vigorous exercise or abdominal or chest surgery were excluded from the study.

### 2.4. Sampling Technique

The sample consisted of all flour mill workers in the mill and matched non-flour mill workers in Addis Ababa, Lideta subcity. Lideta subcity was selected randomly by lottery method of the ten subcities. Lideta subcity contains 10 woredas and 76 flour mills. Among 10 woredas 4 woredas were selected by random sampling. There are 30 flour mills in the 4 woredas and 24 flour mills in the 4 woredas were selected for this study until sample size was saturated. Non-flour mill workers in Lideta subcity matched for age, sex, height, and weight to flour mill workers were selected as controls. Flour mill workers and controls age was 18-64 years selected by convenient sampling technique. Selected flour mill workers and controls fulfilling eligibility criteria were interviewed and anthropometry was measured and finally spirometry was done.

Each participant was informed about the objective of the study and the benefit associated with the study immediately before sample collection. Millers who volunteered to participate in the study have answered questions in the questionnaire, which were relevant to their sociodemographic information. A structured questionnaire was prepared in English and translated into Amharic language and was retranslated back to English by a linguist to ensure consistency. Training was given to the data collectors regarding the objectives of the study and ways of administering the questionnaire by the principal investigator. The prepared questionnaire was pretested prior to the actual data collection on seven (7) informants that were not included in the main survey.

#### 2.4.1. Anthropometric Measurements

Height and weight of the study participants were measured. The height was measured without shoes by the use of a meter rule and approximated to the nearest one cm. Weight was also measured using weighing scale (nearest to one kg), with light clothing and without phones or any encumbrance that could alter their appropriate weight. Body mass index (BMI) was calculated through this formula: weight/squared height (kg/m^2^)

#### 2.4.2. Spirometric Measurement

Digital pocket-sized Spiro Pro (JEAGER, Germany) was used to measure pulmonary function indices. Spirometric tests in both exposed and control subjects were done based on ATS guideline. Before every test, the mouthpiece was disinfected in standard solution and then attached to the spirometer. Subjects were told to put the nosepiece on the nose to prevent air blowout. Then, they were also instructed to breathe in fully until their lung filled maximally. Precaution was taken to avoid leakage from mouth pieces. The subjects expire forcefully and as fast and complete as possible until there is no more air left to expel. Spirometric measurements were performed in sitting position and at a fixed time of the day at room temperature (20-25°C), to minimize diurnal variations. The measurements were done in three trials, and one of the three trials, which was the best, was taken and interpreted. Prior to the actual tests, calibration with one-liter syringe and familiarization steps were done.

#### 2.4.3. Data Analysis

Data was edited, coded, entered, and analyzed by SPSS version 20. Independent sample t-test was used for the comparison of the actual value mean respiratory score of flour mill workers and controls. Bivariate analyses were computed to test whether there was association between FVC, FEV_1_, FEV_1_/FVC, PEFR, and FEF_25_% _−75_% and duration of exposure. Binary logistic regression and odds ratio were used to analyze and estimate the prevalence of respiratory symptoms. Variables having P value <0.05 are considered as statistically significant.

#### 2.4.4. Ethical Consideration

The study was carried out after ethical clearance and approval was obtained from research committee of the Department of Physiology, Addis Ababa University. The study participants were briefed about the objective and procedures of the study. Thereafter, both verbal and written informed consents were obtained from those volunteers and were selected for the study. Confidentiality of information was maintained by excluding personal identifiers.

## 3. Results

### 3.1. Sociodemographic Characteristics

All workers included in the study were males. The age of the flour mill workers ranged between 18 and 43 years with a mean of 27.37±6.71 years while the age of the comparison group ranged between 18 and 43 years with a mean of 28.00±5.33 years (Table 1).

### 3.2. Use of Personal Protective Equipment

**Figure 1:**
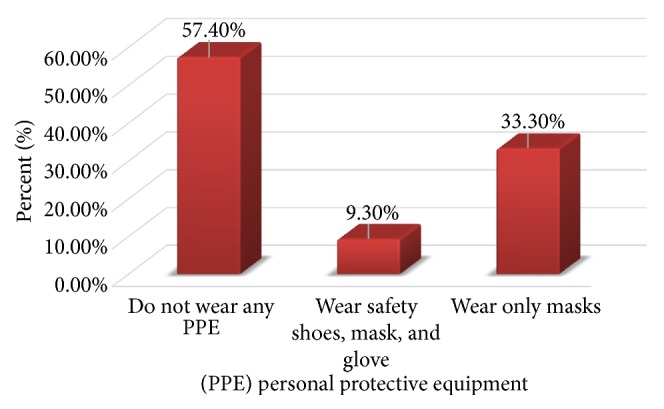
Bar chart indicating the use of personal protective equipment.

Personal protective devices used by the workers were investigated in the study area. Among workers, 57.4% (n= 31) do not wear any equipment, 9.3% (n=5) wear safety shoes, masks, and gloves, and 33.3% (n=18) wear only masks (Figure 1). We observed that there is no sufficient personal protective device in the workplace and the workers do not wear the equipment timely even if the material is available. Among the study subjects, 92% of the informants agreed with the idea that all flour mill workers should wear personal protective devices.

### 3.3. Anthropometric Measurements

Anthropometric parameters of the study subjects in terms of demographic variables demonstrate the comparison between the flour mill workers and their matched control subjects. There were no significant differences between the means of anthropometric parameters: in terms of age, weight, height, and BMI between the groups. The statistical comparison of the matching variables (age, height, weight, and BMI) shows no difference between the two groups (Table 2).

### 3.4. Respiratory Symptoms

The percentage prevalence of dry cough, productive cough, wheeze, and breathlessness was 27.7%, 11.1%, 14.8%, and 16.6% for exposed informants, respectively, and 9.3%, 5.6%, 3.7%, and 7.4% for control subjects, respectively (Table 3). These values were higher among exposed workers as compared to control groups and the difference was statistically significant for dry cough (p<0.05).

Binary logistic regression was used to compare the respiratory symptoms and measure the strength of the association. The odds of dry cough in flour mill workers exposed to flour dust association were found to be statistically significant (p<0.05).

### 3.5. Pulmonary Function Tests

Percentage predicted values of exposed and control groups were indicated (Table 4). There was statistically more significant reduction in the percentage predicted of the exposed group than in healthy control group. Percentage predicted values of control group have largest score when compared to that of the exposed group. The difference was statistically significant (p<0.05). However, for FEV_1_/FVC difference is not significant (p>0.05).

The independent sample t-test (Table 5) for equality of means between two independent groups showed that there is a significant reduction in pulmonary function values of exposed group compared to that in the control (p<0.05). The comparison of the mean spirometric indices of flour mill and controls groups (mean ±SD) indicates FVC, FEV_1_, and PEFR are highly significant (P<0.001) and FEF_25_% _−75_% are significant (P<0.01). The forced expiratory flow rate at the middle fifty percent (FEF_25_%  _−75_%) of the exposed group was lower (3.87L/s) than that of the controls (4.60 L/s). This reduction in flow rate was statistically significant (P <0.01). The PEFR in flour mill workers of the exposed group was lower (5.43 L/s) than that of the controls (7.87 L/s); this reduction was statistically significant (P<0.001).

There was slight reduction in pulmonary function indices (FVC, FEV_1_, and FEV1/FVC, PEFR and FEF_25_%  _−75_%) as the duration of exposure increases. Both pulmonary function indices and duration of exposure move in opposite direction. Based on their service years (1-12 years) duration of exposure and pulmonary function parameters relationship were determined. Bivariate correlation result showed negative correlation between FVC and duration of exposure which was found to be statistically significant (r= -.417, p <0.001). Pearson‘s correlation coefficient was used to quantify the degree of linear relationship between pulmonary function indices (FVC, FEV_1_, FEV_1_/FVC, PEFR, and FEF_25_%  _−75_%) and duration of exposure (Table 6).

### 3.6. Pulmonary Function Status

Flour mill workers were suffering from both obstructive and restrictive disorder. Based on PFTs interpretation results indicate reduction in the pulmonary function efficiency among the flour mill workers. Airflow obstruction which reduces dynamic airway collapse makes expiration difficult. FEV_1_/FVC ratio <0.7 and FEV_1_ value <80% percentage predicted are considered as obstructive pattern. Restrictive defect needs large amount of elastic work to inflate the lung, which makes inspiration difficult. FEV_1_ /FVC ratio >0. 7 and FVC value <80% of percentage predicted are restrictive pattern.

**Figure 2:**
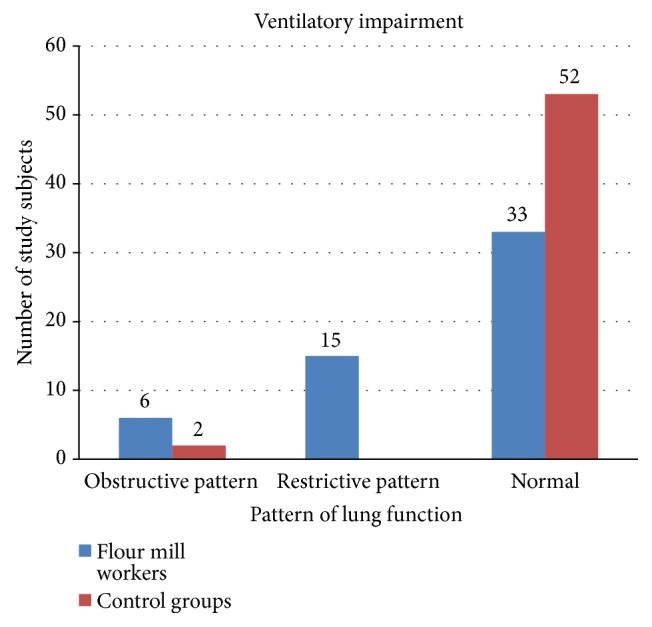
Bar chart showing the number of obstructive, restrictive, and normal patterns of lung function in controls and flour mill workers.

Among 54 flour mill workers (n=15), 27.7% developed restrictive and (n=6) 11.1% developed obstructive type of lung disorder. The rest of the study subjects 61.1% (n=33) had normal pulmonary function. Two subjects (3.7%) in the control group developed obstructive type of lung disorder and the rest of the subjects had normal pulmonary function status (Figure 2).

## 4. Discussion

The present study revealed the prevalence of respiratory symptoms of dry cough (27.7% vs. 9.3%), productive cough (11.1% vs. 5.6%), wheeze (14.8% vs. 3.7%), and breathlessness (16.6% vs. 7.4%) in flour mill workers and controls, respectively. In this study, the higher prevalence of respiratory symptoms in flour mill workers may be due to prolonged exposure of flour dust, unhygienic conditions, and poorly ventilated workplaces of the study areas as compared to the controls. A study by Wagh and his colleagues on the influence of flour dust on flour mill workers compared to the nonexposed controls found that flour mill workers had three times shortness of breath and developed four times frequent coughing compared to controls [11]

Similar findings were reported in Egypt [7] on the effects of exposure to flour dust on respiratory symptoms and lung function of bakery workers. The study showed that flour mill workers had cough, wheezing, and shortness of breath. Another study in Khartoum (Sudan) also showed that daily work related respiratory symptoms were significantly increased in cases compared to controls [5]. Study conducted in India showed the prevalence of respiratory symptoms and disorders among rice mill workers developing phlegm, dyspnea, chest tightness, and cough due to organic dust exposure [15].

The present study investigated the prevalence and the type of pulmonary impairment observed among the flour mill workers. We found 27.7% (n=15) of the workers develop restrictive type of lung disorder while 11.1% (n=6) develop obstructive type of lung disease. On the other hand, there were only two control subjects (3.7%) that showed an obstructive type of lung disorder. Hence, the prevalence of obstructive and restrictive lung diseases was higher in exposed group than in control group. The plausible explanation for the increased prevalence of restrictive lung impairment in exposed group is mainly due to flour dust that reacts with lymphoid and connective tissue in the terminal and respiratory bronchioles and interstitial inflammatory cells.

The results of spirometry done by [16] showed that both controls and mill workers develop lung abnormality. Flour mill workers had five times lung function abnormalities compared to the controls. Similar study showed that workers exposed to flour dust have developed chronic bronchial irritation, which is responsible for the restrictive plus obstructive type of diseases. A study conduct by [17] showed that ventilatory impairments of lung function of flour mill workers were pure restriction and mixed type. Similarly, a study by [11] found that prevalence of ventilatory impairment showed airflow obstruction and restrictive defect were much higher in cases than in controls. This means our study revealed that all lung volumes and flow rates, i.e., FVC, FEV_1_, FEV_1_ /FVC, FEF_25_% _−75_%, and PEFR, showed statistically significant reductions in the mean values in the flour mill workers as compared to their matched controls.

The findings of the present study were in line with others [17] who reported a decrease in PEFR and association between duration of exposure and pulmonary function change. The longer summative time of exposure to flour dust was associated with reduced spirometric values compared to the controls [18]. Highly significant decrement in PEFR in flour mill workers may suggest the involvement and impairment of the larger airways. This may be due to irritative effect of flour dust causing hypertrophy of mucosal cells with increased secretion of mucous, a cause of obstruction to the exhaled air. PEFR is persistently low and represents collapsing of large airways.

A study by [19] also observed a significant decline in the lung function parameters that include FVC and FEV_1_, in workers exposed to flour dust compared to the control group, suggesting that flour dust persistently affects the pulmonary function parameters. The present study is also consistent with another investigation that showed flour dust can adversely affect FVC, FEV_1_, and FEV_1_/FVC [20]. We found significant negative correlation for FVC, FEV_1_, PEFR, and FEF_25_% _−75_% and duration of flour dust exposure. This is in line with other findings that long-term exposure to flour dust decreased FVC, FEV_1_, PEFR, and FEF_25_%  _−75_% values [13, 17, 21]. Reduction in FEF_25_% _−75_% value in exposed workers may be due to the obstructive pattern of the medium and smaller airways in the flour mill workers.

Similar studies conducted in Nigeria [8] showed that flour millers recorded significantly lower mean lung functions compared with control subjects with an increased risk of developing abnormalities of lung functions and there is an association on the overall mean pulmonary function to the duration of exposure of the flour mill workers.

We found that majority of the workers do not wear any equipment, some workers wear safety shoe, masks, and gloves, and 33.3% wear only masks. Another similar study found that some workers used a mask [16].

## 5. Limitations of the Study

This study failed to estimate the concentration of organic flour dust in the working area and was unable to determine subclinical cardiopulmonary and other systemic diseases that may affect the result of the study in both cases and controls.

## 6. Conclusions

Percentage prevalence of respiratory symptoms was higher among exposed workers as compared to control groups and the difference was more vivid for dry cough. Flour mill workers showed significantly reduced values in the pulmonary function tests (FVC, FEV_1_, FEV_1_ /FVC, PEFR, and FEF_25_% _−75_%) and lung efficiency due to excessive exposure to fine organic flour dust as compared to the controls. There is an inverse relationship between duration of exposure and respiratory function which means that when the duration of exposure increases, there is a deterioration of pulmonary function indices. Flour mill workers develop both restrictive and obstructive types of lung disorder. Personal protective devices were not available at workplaces and the workers usually do not wear the materials.

## Figures and Tables

**Table 1:** Sociodemographic information of flour mill workers and controls.

Variables	Descriptions	Flour mill workers (n=54)	Control Group(n=54)	P –value
Age range(in years)	18-23	(n=20)37.03%	(n=13) 20.04%	
	24-28	(n=15) 27.7%	(n=20) 37.03%	0.591
	29-33	(n=7) 12.9%	(n=12) 22.2%	
	34-38	(n=6) 11.1%	(n=7)12.9%	
	39-43	(n=6)11.1%	(n=2) 3.7%	

Educational status	Illiterate	(n= 9) 16.6%	0(0)	
	Can write and read	(n= 19) 35.1%	(n=4) 7.4%	
	Grade 8 complete	(n= 15) 27.7%	(n=1) 1.8%	<0.01
	Grade 10 complete	(n= 8)14.8%	(n=15) 27.7%	
	Grade 12 complete	(n= 1) 1.8%	(n= 18) 33.3%	
	Diploma	0(0)	(n= 6)11.1%	
	1^st^ degree	(n=2) 3.7%	(n=10)18.5%	

Marital status	Single	(n=37) 68.5%	(n= 35) 64.8%	
	Married	(n= 14) 25.9%	(n= 17) 31.4%	0.868
	Divorced	(n=3) 5.5%	(n=2) 3.7%	

**Table 2:** The anthropometric measurements for flour mill workers and control groups by independent sample t-test.

Parameters	Exposed group( n=54) Mean ±SD	Non exposed group (n=54) Mean ±SD	P-value
Age (years)	27.37±6.71	28.00±5.33	0.591
Weight (kg)	59.72±7.76	61.91±6.39	0.113
Height (meter)	1.66±0.07	1.68±0.05	0.388
BMI (kg/m^2^)	21.42±2.25	21.95±1.82	0.185

**Table 3:** Respiratory symptoms of exposed and nonexposed subjects including OR and 95% CI.

Respiratory symptoms		Prevalence % (n=54)	OR	P-value	95% CI	
					Lower	upper
Dry Cough	case	27.7%(n=15)				
	Control	9.3%(n=5)	3.58	.027^*∗*^	1.16	11.65

Productive cough	case	11.1%(n=6)				
	Control	5.6%(n=3)	1.76	.467	0.39	7.98

Wheeze	case	14.8% (n=8)				
	Control	3.7%(n=2)	4.25	.084	0.83	21.88

Breathlessness	case	16.6 %(n=9)				
	Control	7.4%(n=4)	2.80	.115	0.78	10.09

*Symbol*. OR = odd ratio and CI = confidence intervals.

**Table 4:** Percentage predicted lung function tests of exposed and control groups.

Pulmonary function test Parameters	Groups( case n=54 and control n=54)	Mean ±SD	p-value
FVC(L)	Case	88.59±19.05	0.000^*∗∗*^
	Control	107.98±14.05	

FEV_1_ (L)	Case	87.80±20.47	0.000^*∗∗*^
	Control	108.93±16.98	

FEV_1_/FVC	Case	98.56±15.99	0.271
	Control	102.01±16.35	

PEFR(L/s)	Case	59.76±25.37	0.000^*∗∗*^
	Control	83.00±27.32	

FEF_25_% _−75_ % ( L/s)	Case	88.83±35.08	0.001^*∗*^
	Control	115.09±31.34	

**Table 5:** The comparison of the actual value mean spirometric indices of exposed and nonexposed groups by independent sample t-test.

PFTs Parameters	Flour mill workers n=54 and controls n=54)	Mean ±SD	p-value
FVC(L)	Flour mill workers	*4.25±0.93*	p<0.001
	Control	*5.30±0.71*	

FEV_1_ (L)	Flour mill workers	*3.46±0.86*	p<0.001
	Control	*4.50±0.72*	

FEV_1_%	Flour mill workers	*81.93±12.74*	* p>0.05*
	Control	*83.40±12.50,*	

PEFR(L/s)	Flour mill workers	*5.43±2.43*	* p<0.001*
	Control	*7.87±2.53*	

FEF_25_% _−75_ % (L/s)	Flour mill workers	3.87±1.61	p<0.05
	Control	4.60±1.60	

**Table 6:** Pearson correlation coefficient results between pulmonary function parameters and duration of exposure.

Variables	“r” –value	P-value
FVC	-0.417	0.000^*∗∗*^
FEV_1_	-0.359	0.000^*∗∗*^
FEV_1_/FVC	0.060	0.535
PEFR	-0.236	0.014^*∗*^
FEF_25_% _−75_ %	-0.156	0.123

## Data Availability

The data used to support the findings of this study are available from the corresponding author upon request.

## Acronyms and Abbreviations

ATS: American Thoracic Society
BMRC: British Medical Research Council Questionnaire
BMI: Body mass index
FVC: Forced vital capacity
FEV_1_: Forced expiratory volume in one second
FEV_1_/FVC: Percentage of the FVC
FEF_25_% _−75_%: Forced expiratory flow rate at the middle part of FVC
NIOSH: National Institute of Occupational Safety and Health
OAD: Occupational asthma disease
PPE: Personal protective equipment
PFTs: Pulmonary function tests
PEFR: Peak expiratory flow rate
SPSS: Statistical Package for Social Science.
